# Editorial: Genetic control of insect pest species—achievements, challenges, and perspectives

**DOI:** 10.3389/fbioe.2023.1208677

**Published:** 2023-05-05

**Authors:** Irina Häcker, Detlef Bartsch, Amanda Choo, František Marec

**Affiliations:** ^1^ Department of Insect Biotechnology in Plant Protection, Justus-Liebig University Giessen, Giessen, Germany; ^2^ Federal Office for Consumer Protection and Food Safety (BVL), Braunschweig, Germany; ^3^ School of Biological Sciences, University of Adelaide, Adelaide, SA, Australia; ^4^ Biology Centre, Czech Academy of Sciences, Institute of Entomology, České Budějovice, Czechia

**Keywords:** insect pest management, new genetic technologies, sterile insect technique, biological control, environmental-friendly

Insect pests cause billions of dollars of losses in agriculture and livestock and hundreds of millions of disease cases every year due to the transmission of pathogens and parasites. Today, insecticide-based applications are still the most widespread strategy for controlling insect pests and disease vectors, and in many cases are the only effective solution available. At the same time, however, an increasing development of resistance to the main substance classes is being observed worldwide and in many different species. Moreover, the unacceptable impacts of insecticides on human health, non-target species, and the environment and biodiversity has become a major concern in recent decades. Alternative control approaches for insect pests that are effective, sustainable and species-specific are therefore in high demand.

Genetic control is a type of biological control and a promising approach to regulate insect pest populations in a species-specific manner. It is based on targeting the reproductive capacity of the target pest species to reduce population size to non-critical levels. The best known and also very successful genetic control strategy is the Sterile Insect Technique (SIT), which entails the continuous mass-release of irradiation-sterilized males of a given species to produce infertile matings in the field, leading to the decline in the target population over time. To date, SIT is only available for a few species, as its transfer to new target species is challenging and time consuming. Key aspects of this classical SIT and challenges in applying it to new pest species include mass rearing of target species, mass removal of female insects prior to irradiation and release, the sterilization procedure, and the biological quality control of the sterile insects produced.

Besides this classical SIT strategy, current research efforts are also focused on the development of genetic control approaches based on transgenic, symbiont-mediated, or gene-drive strategies. Modern genetic technologies offer new solutions for the improvement of existing genetic control strategies and insect strains, for faster and easier transfer of existing strategies to new target species, and also for the development of new genetic control approaches. Publications within this Research Topic address pressing questions and challenges related to the genetic control of insect pests.

Several control approaches rely on the manipulation of genes of the sex determination pathway, for example, to create strains in which females carry a dominant lethal or strains in which the sex ratio is skewed towards males, both with the aim of suppressing a target population upon release of such insects. Such strategies require detailed knowledge of the sex determination pathway and the genes involved. In their review “Manipulation of Insect Sex Determination Pathways for Genetic Pest Management: Opportunities and Challenges,” Sidall et al. highlights how knowledge of sex determination in target pest species is essential for all phases of development of control technologies. Belavilas-Trovas et al. address the reproductive capacity of mosquito females from a completely new angle by studying the involvement of long noncoding RNAs in *Aedes albopictus* reproduction, which could open a new avenue for insect pest management

The important area of sex separation (“sexing”) for male-only releases was addressed within this Research Topic. Sexing may involve the removal of females during mass rearing by killing them or by sex sorting based on a male- or female-specific marker. Yamamato et al. produced several sexing strains in the agricultural pest *Drosophila suzukii* carrying a double female-specific lethal construct at different genomic positions to eliminate females before irradiation and evaluated their sexing performance as well as general fitness and male sexual competitiveness. One of their aims was to generate strains that can be tested against strains with different genetic backgrounds and they identified promising lines that can be used in such population suppression experiments.

Once such sexing strains are considered for application in the field, their functionality and performance in the local genomic background of the release area must be verified. Augustinos et al. evaluated the stability of *Aedes aegypti* sexing strains carrying a red marker and a recombination-suppressing inversion in six different genomic backgrounds. The same sexing traits were introgressed into the genomic background of the Northern areas of Pakistan’s KP Province and tested for their genetic stability, biological quality, and their potential to be used for SIT applications against *Ae. aegypti* populations in Pakistan by Misbah-ul-Haq et al.

Another key aspect of genetic control strategies is the biological quality of the insects produced. The success of genetic control programs depends heavily on the dispersal rate, longevity, and mating success of released males. These parameters are influenced by conditions and procedures of mass rearing and irradiation, as well as by the transport of insects to release sites. Therefore, quality assessment and improvement of the insect strains and produced males are important aspects to ensure successful application. Several publications on this Research Topic address these important questions.

In order to reliably evaluate and, more importantly, compare the quality of insect strains and males produced, standardized and universally applied methods are needed. To test the flight ability of mosquito males, IAEA/FAO developed a flight test device that was further optimized and standardized in the study by Maiga et al. to ensure quality control of mosquito males. Yamada et al. used this flight test device to investigate how immobilization of mosquitoes during irradiation affects one of the standard parameters for male quality, flight ability. Mosquitoes are commonly immobilized during irradiation by chilling or anesthetics (nitrogen) to reduce damage caused by movement in confined spaces. They also tested longevity and evaluated the irradiation dose response in *Aedes* mosquitoes in combination with chilling or nitrogen exposure.

Quality effects in a transgenic sexing strain was also examined. One technology used to create sexing strains in several insect species is the tetracycline-off system. It consists of a lethal gene cassette whose expression can be controlled by the presence of the antibiotic tetracycline or doxycycline. Yan et al. studied the parental and transgenerational effects of these antibiotics in combination with the genomic position of the transgene in *D. suzukii*.

Insect performance must always be tested outside the laboratory setting and standard test conditions, i.e., in the field. Velo et al. conducted mark-release-recapture studies on irradiation-sterilized *Aedes albopictus* males produced in Italy and shipped to Albania to estimate, under field conditions, their dispersal capacity, probability of daily survival and competitiveness, and target population size in a highly urbanized area. Using sterile males from the same production facility, Balestrino et al. investigated how environmental factors and weather parameters affect the dispersal rate of released males in Italy. In another publication, Balestrino et al. investigated the effect of irradiation from a completely different angle: the effects of irradiation on vector competence. They compared the virus load and transmission efficiency in two *Aedes* species, *Ae. aegypti* and *Ae. albopictus*, with or without the irradiation dose of 40 Gy.

Gene drives offer another option to suppress insect pest populations, e.g., by driving a lethal trait into the natural population. Because this trait spreads with super-Mendelian inheritance, only small release numbers are required, largely eliminating the need for mass rearing when gene drive approaches are used. Moreover, gene drives can be used to replace natural populations, e.g., by introducing mosquitoes that are refractory to disease transmission, thereby maintaining the natural role of mosquitoes in food webs but abolishing vector capacity. In recent years, many different gene drive architectures have been developed, each bringing its own advantages and challenges. Gene drives can be powerful transformative technologies, and thus their potential application in the field also raises various concerns about how precisely such technologies can be controlled spatiotemporally, and what unintended consequences might result from their use. Verkuijl et al. address these Research Topic in their review entitled “Challenges in Developing Efficient and Robust Synthetic Homing Endonuclease Gene Drives,” while Chennuri et al. describe the current state of the art genetic approaches for controlling CRISPR-based autonomous homing gene drives. Gene drive constructs tend to be large and complex and consist of multiple different elements, making it difficult to generate new gene drives and test the behavior of the different functional modules. Integral gene drives, characterized by a modular design, have been developed, allowing step-wise testing of the gene drive components before they become fully autonomous drives. In the current study Nash et al. took the next step and constructed an autonomous integral drive using intronic guide RNAs.

In addition to safety concerns regarding gene drive releases, there are also ethical questions surrounding the advancement of the technology. The development and release of gene drives involves many different stakeholders, and one important Research Topic is the co-development of the technology with local stakeholders and communities and the reduction of asymmetry between developers and end-users. Kormos et al. discuss Research Topic with true stakeholder involvement and co-development, particularly with respect to stakeholders in low-income countries.

While new genetic tools and technologies promise solutions to many pressing problems in the sustainable and environmentally friendly control of insect pests, any release of genetically modified organisms into the environment requires a thorough evaluation and risk assessment of each genetically modified insect strain that is to be released. Alcalay et al. investigate the probability of an X-shredder relocation from an autosome to the Y chromosome. X-chromosome shredding is a mechanism to induce sex ratio distortion by biasing spermatogenesis towards Y-bearing gametes. Relocation of the X-shredder to the Y-chromosome could therefore result in invasive meiotic drive element.

Finally, despite rapid advances in genetic engineering in many different insect species and the rapid adaptation of new technologies such as CRISPR/Cas genome editing, not all insect orders are equally amenable to these technologies. One order that is lagging behind is the order Hemiptera. Pacheco et al. review the progress, challenges, and perspectives of gene editing and genetic control of hemipteran pests.

